# No Evidence of Enemy Release in Pathogen and Microbial Communities of Common Wasps (*Vespula vulgaris*) in Their Native and Introduced Range

**DOI:** 10.1371/journal.pone.0121358

**Published:** 2015-03-23

**Authors:** Philip J. Lester, Peter J. Bosch, Monica A. M. Gruber, Eugene A. Kapp, Lifeng Peng, Evan C. Brenton-Rule, Joe Buchanan, Wlodek L. Stanislawek, Michael Archer, Juan C. Corley, Maitè Masciocchi, Annette Van Oystaeyen, Tom Wenseleers

**Affiliations:** 1 Centre for Biodiversity and Restoration Ecology, Victoria University of Wellington, Wellington, New Zealand; 2 Centre for Biodiscovery, Victoria University of Wellington, Wellington, New Zealand; 3 Department of Biology, University of Iowa, Iowa City, Iowa, United States of America; 4 Walter and Eliza Hall Institute of Medical Research and University of Melbourne, Melbourne, Australia; 5 Investigation and Diagnostic Centre—Wallaceville, Ministry for Primary Industries, Upper Hutt, New Zealand; 6 York St. John University, Lord Mayor’s Walk, York, England; 7 CONICET and Grupo de Ecologia de Poblaciones de Insectos, INTA EEA Bariloche, Argentina; 8 Laboratory of Socio-Ecology and Social Evolution, University of Leuven, Leuven, Belgium; National Research Laboratory of Defense Proteins, REPUBLIC OF KOREA

## Abstract

When invasive species move to new environments they typically experience population bottlenecks that limit the probability that pathogens and parasites are also moved. The invasive species may thus be released from biotic interactions that can be a major source of density-dependent mortality, referred to as enemy release. We examined for evidence of enemy release in populations of the common wasp (*Vespula vulgaris*), which attains high densities and represents a major threat to biodiversity in its invaded range. Mass spectrometry proteomic methods were used to compare the microbial communities in wasp populations in the native (Belgium and England) and invaded range (Argentina and New Zealand). We found no evidence of enemy release, as the number of microbial taxa was similar in both the introduced and native range. However, some evidence of distinctiveness in the microbial communities was observed between countries. The pathogens observed were similar to a variety of taxa observed in honey bees. These taxa included *Nosema*, *Paenibacillus*, and *Yersina* spp. Genomic methods confirmed a diversity of *Nosema* spp., Actinobacteria, and the Deformed wing and Kashmir bee viruses. We also analysed published records of bacteria, viruses, nematodes and fungi from both *V*. *vulgaris* and the related invader *V*. *germanica*. Thirty-three different microorganism taxa have been associated with wasps including Kashmir bee virus and entomophagous fungi such as *Aspergillus flavus*. There was no evidence that the presence or absence of these microorganisms was dependent on region of wasp samples (i.e. their native or invaded range). Given the similarity of the wasp pathogen fauna to that from honey bees, the lack of enemy release in wasp populations is probably related to spill-over or spill-back from bees and other social insects. Social insects appear to form a reservoir of generalist parasites and pathogens, which makes the management of wasp and bee disease difficult.

## Introduction

The enemy release hypothesis proposes that invasive species become abundant in an introduced range because of the absence of natural enemies such as pathogens and parasites [1,2]. Pathogens and parasites are rarely ubiquitous within any population. Consequently, when an individual or only a few individuals of an invasive species are moved to a new environment they experience a bottleneck that could potentially limit the probability that pathogens or parasites are also moved to the new range. The invasive species may thus be ‘released’ from biotic interactions that can be a major source of density-dependent mortality. A recent review found similar numbers of studies supporting as questioning the hypothesis [3]. This review did find significant evidence to support aspects of the enemy release hypothesis including that invasive species experience less infestation with enemies in their exotic compared to native range.

The common wasp (*Vespula vulgaris* (L.)) is an invasive species native to and widespread in Eurasia [4,5]. In New Zealand these wasps can reach the world’s highest known densities of up to 370 wasps per m^2^ of tree trunk [6] and 34 nests per ha [7]. These high densities are the driver of substantial ecological impacts, which include high predation rates on invertebrates and the domination of food resources [8,9]. Populations of these wasps in Argentina and New Zealand appear to have originated in Western Europe, with populations in the invaded range exhibiting high genetic similarity to those from Belgium and the United Kingdom [10]. Densities of common wasps within the native range fluctuate substantially. Years of high abundance are frequently followed by years of scarcity, with queen productivity varying by a factor of 100 between different nests and years [11]. These results suggest some form of endogenous density-dependence, which in addition to exogenous factors such as climate can promote high wasp abundances [11,12]. Population dynamics within the introduced range show much less fluctuation [13]. This difference in abundance and population fluctuation might be related to several factors including food availability and the abundance of natural enemies such as pathogens and parasites.

The diversity and potential regulatory role of pathogens and parasites in social insects has been highlighted by "colony collapse disorder" in honey bee populations. The exact causes of this disorder in honey bees are unknown, but likely involve a combination of several pathogens or parasites [14]. In addition, the beneficial gut bacterial communities of bees are gaining increasing attention as likely mediators of pathogen effects [15]. Pathogens and mutualistic microbes alike may be transferred horizontally, even between species, by behaviours such as feeding on the same food source (e.g. nectar [16]) and hive robbing [17]. Rose et al. [18] found records of 50 fungal, 12 bacterial, five to seven nematodes, four protozoans, and two viral species from wasps in the genera *Vespula*, *Vespa*, and *Dolichovespula*. More recent work has reported additional pathogens and parasites in Vespulid wasps (e.g. [19,20]).

Mass spectrometry based proteomics is emerging as an important tool for molecular and cellular biology, as it can identify and quantify hundreds to thousands of proteins from complex samples [21] including proteins from parasites and pathogens. As Bromenshenk et al. [22] suggest, proteomics has the advantage that the identification and classification of microorganisms from the environment is unrestricted by the need for amplification, probes, or primers. In addition, this approach allows for the detection, quantification, and classification of fungi, bacteria, and viruses in a single analytical pass [23,24]. However, much like DNA-based methods, pathogen or parasite identification is limited by the quality of the databases [25]. Care should be taken with protein identifications produced from expressed sequence tags, as they do not represent the entire coding sequence from a gene or protein. Consequently, identifications derived from any high throughput method need to be judged carefully [26]. Ideally, an additional method of confirmation should be used in order to provide additional confidence regarding the identity of potential pathogens and parasites.

**Fig 1:**
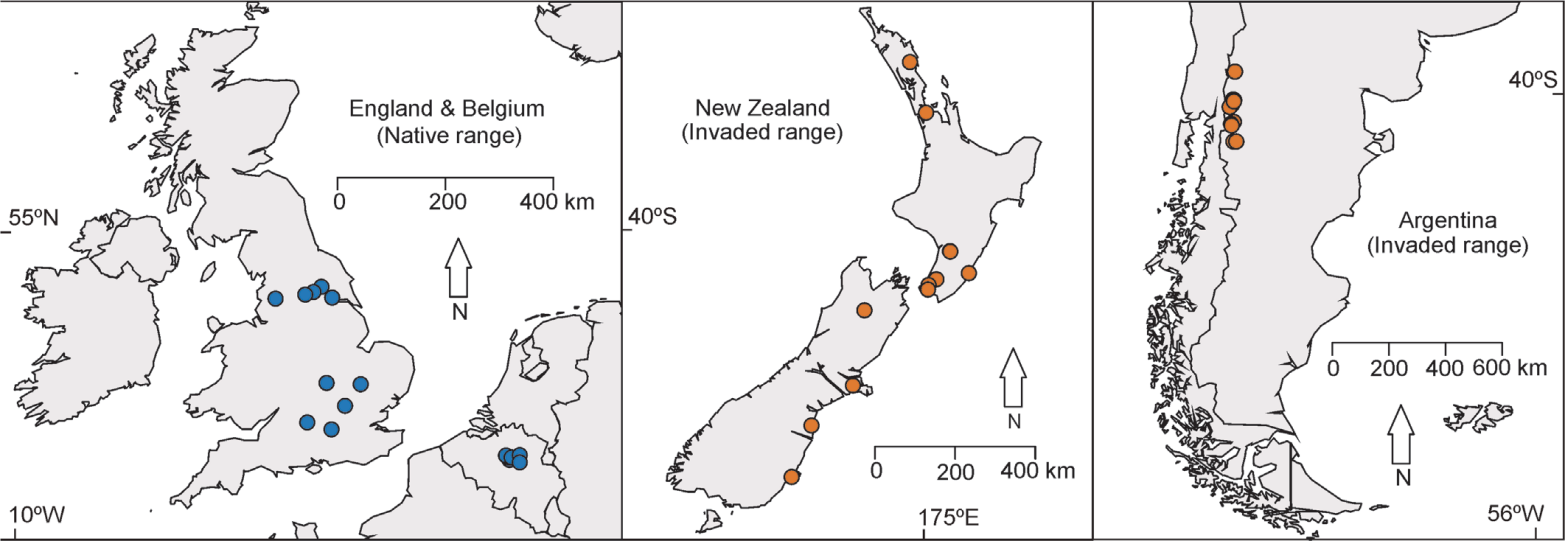
Sample locations for common wasps from the native (England and Belgium) and invaded range (New Zealand and Argentina). Twenty adult *V*. *vulgaris* worker wasps were collected from each of the four countries. In some cases, multiple wasps were collected in the same area, but never from the same nest. For Argentina, the restricted sampling area represents the latitudinal limits of their distribution at the time of sampling in 2013. Common wasps are distributed throughout New Zealand.

Countries such as New Zealand have no native social wasps or bees, and have been the recipient of only a limited number of invasive wasp propagules [10]. We therefore predicted a reduced diversity of parasites and pathogens in the invaded range of the wasps compared to their native range, effectively testing a key component of the enemy release hypothesis. We first examined published records of bacteria, nematodes and fungi from both *V*. *vulgaris* and *V*. *germanica*. From these published records we tested the hypothesis that the presence or absence of these microorganisms was dependent on region of wasp samples (i.e. their native or invaded range). We next examined for the presence and diversity of pathogens and parasites in two countries of the home range (England and Belgium) and two countries in the invaded range (Argentina and New Zealand) (Fig. 1). Finally, we sought to confirm the presence of several microorganisms identified by proteomics results using PCR methods. The control of the common wasp has been identified as a high priority for conservation in New Zealand [27], and studies such as this may help identify biological control agents for the regulation of wasp densities within their invaded range.

## Materials and Methods

### Enemy release analysis from published data

We examined previously published literature for evidence of enemy release in *Vespula* wasps. Rose et al. [18] conducted a literature survey of possible pathogens of social wasps and obtained information for fungal, bacterial, viral, nematode and trypanosome species found in wasps throughout their native and introduced range. We used records associated with all *V*. *vulgaris* and *V*. *germanica* species (ignoring information for *Vespa* and *Dolichovespula* species). We updated Rose et al. [18] using Web of Science, searching for “*Vespula* and virus”, “*Vespula* and nematode”, “*Vespula* and bacteria”, and “*Vespula* and fungi”. Other specific pathogens potentially not encompassed by these searches were conducted and included “Vespula and *Nosema*” and “Vespula and *Crithidia*”. Searches were undertaken over the period of July to November 2014. All studies either surveying the microbial community or reporting an observation of a microorganism were included, while studies in which wasps were experimentally infected with a pathogen were excluded. Since the publication of Rose et al. [18], microsporidia have been reclassified to fungi rather than protozoa (which are referred to here as trypanosomes), and all previous *V*. *vulgaris* observations in North America were assumed to be the native species *V*. *alascensis* [28] and were thus excluded from this analysis. Within Rose et al. [18] several observations were identified only to the genus level, such as “*Streptococcus* sp.”. We treated all species identifications as a new species, which may have overestimated the microbial community (in comparison, not using these records may underestimate the pathogen community). We also note that these are “possible” pathogens and some of the taxa identified may not be harmful or may even be mutualistic. Any determination of pathogenicity would require experimental work.

Each reported microbial taxon was recorded as present or absent in the native and introduced geographic areas. A binomial generalised linear mixed effects model was then used to test the hypothesis that the presence or absence of the microbial taxa differed between the native and introduced geographic areas. A fully factorial model with the geographic area (native or invaded) and type of taxon (bacteria, fungi, nematodes or viruses) as fixed effects and study as a random effect was initially fitted using the ‘lme4’ package [29] in R [30]. The interaction term was not significant (*P* > 0.050), so we then fitted a model using only the main fixed and random effects. The Intercept of the random effect contributed no variance to the model, so we then fitted a model with fixed effects only. We also used Pearson correlation to test the hypothesis that there would be a positive correlation between the number of studies for bacteria, fungi, nematodes and viruses and the number of microbes or nematodes observed in the native and introduced range.

### Wasp proteomics analysis

Wasps used here were those collected for a separate study on the population genetics of common wasps in their native and invaded range [10]. Twenty individual wasps were taken from each of two countries in the invaded range (Argentina and New Zealand), and from each of two countries within the native range where invasive populations appear likely to have originated (i.e. Belgium and England) (Fig. 1; [10]). The samples of foraging workers or workers from nests were preserved in 90% ethanol prior to being sent to New Zealand, or were frozen immediately. When wasps were taken from nests, only one worker from each nest was used. Wasp samples were in storage < 12 months when used for this analysis. In Belgium, New Zealand, and the United Kingdom the wasps sampling did not require governmental or local authority permission. Wasps in Argentina were collected under permit 1233 from the Administracion de Parques Nacionales. Twenty separate sampling locations were used for Belgium, New Zealand, and the United Kingdom. Only 13 sites were sampled from Argentina, so two wasps were used from the same site in some locations.

Twenty wasps from each location were combined and crushed to a fine powder in liquid nitrogen using a mortar and pestle, resulting in four separate samples corresponding to the four locations. Lysis buffer (7M Urea, 2M Thiourea, 4% Chaps, 0.2% Triton X-100, 0.1% SDS, 40 mM Tris pH 8.5 supplemented with protease inhibitor cocktail (Sigma-Aldrich, P8340, MO, USA), 4–5 w/v) was added to the powdered sample, vortexed at room temperature for 2 h and centrifuged at 15,000 g for 30 min at 4°C. The supernatant containing the wasp proteins was collected and stored at −20°C for further analysis. Protein concentration was determined by Bradford assay (Bio-Rad, Auckland, NZ).

In order to maximise the protein extracted from each sample, we loaded 20 μg of each sample into duplicate wells (i.e. 40 mg total for each sample) in a 4–12% gradient SDS-PAGE gel (NuPage Novex 4–12% Bis-Tris Gels, Life Technologies, Auckland, NZ), which was run at 200 V for 60 min. The gel was removed immediately following electrophoresis and fixed (50% ethanol and 3% phosphoric acid) for 30 min. The gel was washed with milliQ H_2_O and incubated in staining solution (34% methanol, 17% ammonium sulfate and 3% phosphoric acid) for 60 min. A small spatula (approx 10 mg) of Coomassie Brilliant Blue G-250 (#161-0604, Bio-Rad, Auckland, NZ) was added to the solution and left for 3 days to completely stain. Following staining, quick washes with milliQ H_2_O were performed and gels were scanned using the GE ImageScanner III with LabScan software.

The stained gel was washed twice with distilled H_2_O, and the gel lanes were excised. Each gel lane was cut into 20 slices and each slice was diced into approximately 1×1 mm pieces. The gel pieces from duplicate gel lanes of each sample were combined, de-stained in 50% acetonitrile in 50 mM NH_4_HCO_3_, reduced with 10 mM dithiothreitol in 0.1 M NH_4_HCO_3_ for 30 min at 56°C, and alkylated with 55 mM iodoacetamide in 0.1 M NH_4_HCO_3_ for 40 min at room temperature in the dark as previously described [31]. The reduced and alkylated proteins were in-gel digested with trypsin (Roche-modified sequencing grade) in 50 mM NH_4_HCO_3_ at 37°C overnight. The resulting tryptic peptides were sequentially extracted with 2 volumes of 25 mM NH_4_HCO_3_, acetonitrile, 5% formic acid and then acetonitrile again for 15 min at 37°C with shaking. The extracts were pooled and dried under vacuum to approximately 5 μl. The tryptic peptides were purified with PerfectPure C18 tips (Eppendorf AG, Germany) according to the manufacturer’s instruction, and then eluted into 5 μl of 70% ACN, 0.1% formic acid solution. The eluted peptides were raised to 75 μl with 0.1% formic acid (Buffer A of the LC gradient) that are required for two injections for duplicate LC-MS/MS analyses.

The LC-MS/MS was performed in a Dionex UltiMateTM 3000 nano liquid chromatography system coupled with a Linear Trap Quadrupole (LTQ) XL Orbitrap mass spectrometer via a nanospray ion source (Thermo Fisher Scientific, USA). The peptides were separated on a 75 μm ID × 15 cm PepMap C18 column (3 μm, 300 Å, Thermo Fisher Scientific, USA) at a flow rate of 0.3 μL/min using a gradient constructed from 0.1% formic acid (Buffer A) and 0.1% formic acid in 80% acetonitrile (Buffer B): 2–20% B for 10 min, 20–80% B for 60 min, 80–98% B for 5 min, 98% B for 3 min. The eluted peptides were ionised through a PicoTipTM emitter (New Objective, USA) at 1.8 kV. Full MS scan (m/z 200–1850) of the peptide ions was acquired in the Orbitrap with 30,000 resolution in profile mode. The MS/MS scans of the six most intense peptide ions from the full scan were performed using CID in the LTQ (normalised collision energy, 35%; activation Q, 0.250; and activation time, 30 ms) in data-dependent mode. Dynamic exclusion was enabled with the following settings: repeat count, 2; repeat duration, 30 s; exclusion list size, 500; exclusion duration, 90 s. The spectra were acquired using Xcalibur (version 2.1.0 SP1, Thermo Fisher Scientific). The LC-MS/MS experiments were performed in duplicate.

The LC-MS/MS spectra were converted to Mascot generic files (MGFs) for Mascot searches. MGFs contain mass peak lists, and the peak lists were extracted using Proteome Discoverer (version 1.4, Thermo Fisher Scientific). MGFs were searched using the Mascot search algorithm (version 2.5.0, Matrix Science, UK) against two comprehensive sequence databases with no taxonomy applied (NCBI GenBank nr and LudwigNR_Q314) (http://www.ludwig.edu.au/archive/LudwigNR/LudwigNR.pdf). The Mascot search parameters were as follows: carbamidomethylation of cysteine (+57.021 Da) as a fixed modification; N-terminal acetylation (+42.011 Da), N-terminal carbamylation (+43.066), N-terminal Q Gln->pyro-Glu (−17.026) and methionine oxidation (+15.995 Da) as variable modifications. A peptide precursor mass tolerance of 10 ppm, #13C defined as 1, and fragment ion mass tolerance of 0.5 Da were used. The automatic decoy (random) database sequence option was enabled to allow false-discovery rate estimation. Peptide identification and protein inference was performed using Scaffold (version 4.3.4, Proteome Software Inc., USA). The Mascot search result files (.DAT files) derived from the duplicate LC-MS/MS runs of the gel slices of a single SDS-PAGE gel lane were uploaded into Scaffold in combination to generate the overall protein list of a wasp sample. Proteins detected with ≥ 95% probability as assigned by ProteinProphet [32] containing at least one peptide that was detected with ≥ 95% probability as assigned by PeptideProphet [32] were considered as positive identifications. The mass spectrometry proteomics data have been deposited to the ProteomeXchange Consortium (http://proteomecentral.proteomexchange.org) [33] via the PRIDE partner repository with the project number PXD001586 and password W50heXfw.

To determine whether our proteomics assay was likely to have uncovered the diversity of the microbial communities in the four countries sampled, we generated rarefaction curves in R [30] using the ‘vegan’ package [34], and the rarefaction.txt function [35]. It is likely that there was some degree of microbial contamination from recently consumed prey or food items in the gut contents of wasps. Removal of the wasp intestines prior to analysis would, however, have meant we would miss important pathogens [14, 25] and the microbial diversity of the gut contents that can be beneficial to their hymenopteran hosts [15].

Binomial generalised linear models were also used to determine if the presence or absence of the microbial taxa differed among countries and between the native and introduced ranges. Fully factorial models with the geographic area (country or native/ invaded) and type of taxon (bacteria, fungi, nematodes or viruses) as predictors was initially fitted in R [29,30]. The interaction terms were not significant, so we fitted main-effects models for both comparisons by country and by range.

### Phylogenetic comparisons using PCR methods

To establish the phylogenetic position of putative Actinobacteria, *Nosema*, Kashmir Bee Virus and Deformed Wing Virus sequences in our samples we sequenced DNA / cDNA from wasp and bee workers in the native and invaded range. Genomic DNA extractions for *Nosema* and Actinobacteria followed a standard SDS / proteinase-k method. Ethanol preserved material from 5–10 individual wasps from each location were ground together in liquid nitrogen. The ground material (40–60 mg) was incubated in 500μl aqueous buffer containing 10mM Tris-HCl, 50mM NaCl, 10μM EDTA and 1.5gl-1 lysozyme, in a shaking incubator for 1 hour at 37°C. Following that 10% SDS (2% final concentration) and 20μl proteinase-k was added, mixed by inversion and incubated at 55°C overnight, followed by phenol / chloroform purification. The DNA was precipitated with 2.5 volumes 100% ethanol and 0.1 volume sodium acetate at −20° overnight, washed in 70% ethanol, dried and re-suspended in 100μl TE buffer.

We included wasp samples from New Zealand, England and Belgium in the Actinobacteria assay. These samples had been obtained as part of an earlier study [10]. We also included a sample from *Apis mellifera* collected from Wellington, New Zealand (41.289° S, 174.777°E) in February 2014. Actinobacteria-specific 16S primers were used to target the V3 to V5 regions of the 16S rRNA gene (SC-ACT-878 and SC-ACT-235 [36]). Thermal cycling used a touchdown protocol with initial denaturation at 95°C for 2 min; 10 cycles of 95°C for 45 s, 72°C for 45 s (−0.5°C/cycle), 72°C for 1 min; followed by 25 cycles of denaturing at 95°C for 45 s, annealing at 68°C for 45 s, extension at 72°C for 1 min; final extension of 72°C for 10 min.

We included wasp samples from New Zealand, England and Slovakia in the *Nosema* assay. These samples were obtained during an earlier study [10]. We used general microsporidia-specific 16S primers, which amplify the V1–V3 regions of the 16S rRNA gene, to target *Nosema* (V1f and 530r; [37]). Thermal cycling conditions included initial denaturation at 9°C for 2 min; followed by 35 cycles of denaturing at 95°C for 40 s, annealing at 60°C for 40 s and extension at 72°C for 40 s; final extension at 72°C for 10 min.

Each 15 μl PCR consisted of ~20 ng template DNA, 10× PCR Buffer, 0.4 μg ml^-1^ of bovine serum albumin (BSA), 1.5 mM MgCl_2_, 0.1 mM of each dNTP, 0.4 mM forward and reverse primer, 0.4 mM DMSO (for Actinobacteria only) and 0.3 U of Taq DNA Polymerase (Fisher). Amplified products were purified using ExoSAP-IT (US Biochemicals) and sequenced on a 3730 Genetic Analyser (Applied Biosystems).

For the virus assays we included samples from New Zealand from *V*. *vulgaris* and *A*. *mellifera* that were obtained from two colonies in Wellington, New Zealand (41.289° S, 174.777°E). These samples were collected in February 2014 and stored in RNALater. These samples were needed given that RNA degrades in ethanol, which was used to store other samples, but we wanted some indication of virus presence in wasp samples. Total RNA was extracted from wasps and bees using TRIzol LS Reagent (Life Technologies) following the manufacturer’s instructions. Individual samples were homogenised by grinding in a mortar and pestle with phosphate buffered saline. Deformed wing virus (DWV) specific oligonucleotide primer pairs were designed using the Primer3 online design tool (http://simgene.com/Primer3), targeting the DWV helicase protein gene at the position 6453 to 6748 of the DWV genome (GenBank Accession AY292384; [38]). The RT-PCR primer sequences were: DWVrtF 5’-GCAGCTGGAATGAATGCAGAGA -3’ (forward) and DWVrtR 5’-ACGCGCTTAACACACGCAAA -3’(reverse). Primers used for the Kashmir bee virus (KBV) assay were the AKI primers designed to detect the honey bee viruses KBV, Israeli acute paralysis virus (IAPV) and Acute bee paralysis virus (ABPV) in a single assay [39]. The RT-PCR amplifications of the RNA were performed using One Step SYBR PrimeScript RT-PCR kit II (Takara Bio Inc.) according to the manufacturer’s instructions. The thermocycling profile consisted of 42°C for 10 min, followed by 95°C for 10 s and 40 cycles of 95°C, for 5 s and 60°C for 35 s. Melt curve analysis was programmed at the end of the PCR run, from 65–95°C in increments rising by 0.5°C each step and a 5s hold at each degree to determine reaction specificity. The resulting PCR products were sequenced on a 3730 Genetic Analyser (Applied Biosystems). To construct phylogenetic trees to determine the position of the Actinobacteria, *Nosema* and DWV sequences in our samples we used the closest matching results of BLASTn searches of the NCBI (GenBank) nucleotide (nt) database.

We manually checked quality, edited and aligned the sequences using MEGA6 [40]. Sequences of 537 bp were obtained for Actinobacteria, 282 bp for *Nosema*, 101 bp for KBV and240 bp for DWV. BLASTn searches of the NCBI (GenBank) nucleotide (nt) database were used to identify the closest matches to our sequences, which were used to build phylogenetic trees. To determine the most appropriate model of sequence evolution for our datasets, we used Log-likelihood scores (lnL) derived in MEGA6, which also estimated base frequencies, substitution rates, the proportion of invariable sites (I), and the uniformity of substitution rates among sites (G). The best-fitting model for *Nosema* was the general time-reversible model [41,42] with gamma distribution and invariant sites (GTR + G(0.69) +I(0.0); lnL −928.456). For Actinobacteria the best-fitting model was a general time-reversible model with gamma distribution and invariant sites (GTR + G(0.47) +I(200.0); lnL −559.13), and for DWV the best-fitting model was a general time-reversible model with gamma distribution and invariant sites (GTR + G(0.48) +I(200); lnL −550.04). For KBV the best-fitting model was a general time-reversible model (GTR; lnL −195.50). The estimated models were then used to generate Maximum Composite Likelihood (MCL) trees in MEGA6 using nearest-neighbour interchange and a weak branch swap filter, with the level of support assessed with 2000 bootstrap replicates.

## Results

### Enemy release analysis from published data

**Fig 2:**
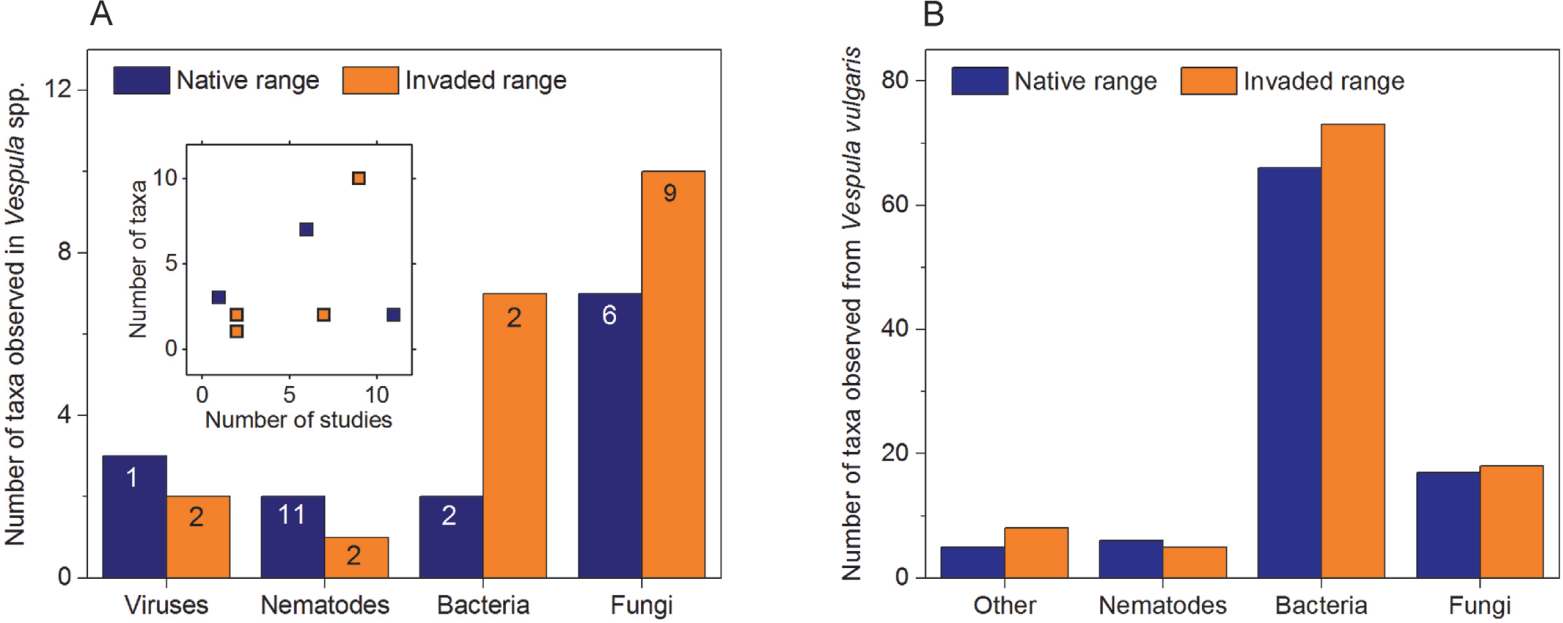
The number of microbial taxa observed from the previously published literature and proteomics methods. (A) The number of microbial taxa observed in published studies examining *V*. *germanica* and *V*. *vulgaris*. The numbers at the top of the bars represent the number of published studies, e.g. there were nine published papers examining fungal in wasps from the invaded range. Inset is a graph showing the non-significant relationship (p≥0.218) between the number of taxa found and the number of studies for each microbial group. (B) Results from our proteomics survey of microbes associated with wasps from the native and invaded range. No viruses were observed in the proteomics analysis. The “other” category is from peptides indicating the presence of taxa including amoeba (*Acanthamoeba* sp.), a protozoan (*Babesia* sp.), and tapeworm (*Taenia* sp.).

We examined published records for records of bacteria, nematodes and fungi from both *V*. *vulgaris* and the related globally invasive species *Vespula germanica* (F.). A total of 31 microbial taxa and three nematode species have been reported from the common wasp (*V*. *vulgaris*) and the German wasp (*V*. *germanica*). The most common records are for fungal species with 17 taxa, followed by bacteria (nine taxa), and viruses (five taxa) (Fig. 2A.). Some of the microbial taxa observed in wasps are known to have pathogenic effects in bees or other insects (e.g. Deformed wing virus, the fungal species *Beauveria bassiana*, and *Nosema* spp.), while other reported taxa may even be beneficial (see S1 Table for a full list of microbial taxa). The number of studies varied substantially between different microbial or nematode taxa. For example, there were 11 reports of nematodes in wasps with the earliest occurring from 1879 [43], which is prior to the discovery and description of viruses. Only three nematode species have been reported from these 11 different studies on *V*. *vulgaris* and *V*. *germanica*. Only three studies were found which observed viruses in these wasps. No work to our knowledge has examined for *Crithidia* spp. trypanosomes, which have been observed in related species such as *V*. *squamosa* [44]. The most extensive study on bacteria associated with these wasps was by Reeson et al. [45] in the introduced range of Australia, which was a study initiated with the goal of developing biological control solutions for *V*. *germanica*.

A binomial generalised linear model found no difference in pathogen presence identified from the historic records between the geographic areas (*F* = 2.025, D.F. = 1, *P* = 0.160), and no difference between pathogen taxa (*F* = 0.000, D.F. = 3, *P* = 1.000; Fig. 2A). We also failed to observe a significant correlation between the number of studies for bacteria, fungi, nematodes and viruses, and the number of microbes or nematodes observed in the native (Pearson *r* = 0.031, *P* = 0.969) and introduced range (Pearson *r* = 0.782, *P* = 0.218).

### Wasp proteomics analysis

We used an LC-MS/MS proteomic analysis to examine for the presence and diversity of pathogens, parasites, and other microbial taxa in two countries of the home range (England and Belgium) and two countries in the invaded range (Argentina and New Zealand). A total of 585 proteins were inferred with ≥95% protein identification probability (see S2 Table for a full list of inferred proteins). Many of the proteins were from hymenopteran species including an insect muscle or actin protein from a leafcutter ant species (*Acromyrmex echinatior*), an ATP synthase protein from honey bees (*Apis mellifera*), and a tropomyosin protein from jewel wasps (*Nasonia vitripennis*). Such proteins were expected given the close phylogenetic relationship of these insects to common wasps.

Of the 585 proteins or peptide sequences inferred via the LC-MS/MS analysis, 135 were identified as being microbial, pathogen or parasite related. Of these, 131 microbial peptides were unique to single taxa: only four microbial taxa had multiple peptides. Peptides from a wide variety of microbial or multi-cellular species were nominally identified, although it is important to note that species-specific identifications are unlikely to be reliable given the limited coverage of the proteome sequence databases. The taxa included 91 bacterial nominal species identifications from 83 different genera. Many of the identified taxa have been previously associated with hymenopteran insects, such as species within the genera *Bacillus*, *Burkholderia*, *Paenibacillus*, *Pseudomonas*, and *Yersina*, and genera within the class Actinobactera. Other microbial species, including *Neorickettsia risticii*, have never been observed in the Hymenoptera, but only in other insect groups. It is possible that such species were misidentified, possibly due to homology, for example from other Rickettsiales species that do occur in the Hymenoptera. We also tentatively identified a total of 25 nominal fungal species belonging to 23 different genera. The identified genera included *Actinoplanes*, *Fusarium*, *Nosema*, *Pseudozyma*, and *Rhodotorula*, which have been associated with hymenoptera. Other fungal genera (e.g. *Emericella*, *Wallemia*) have been previously identified from soil and may represent contaminant from wasp nesting material, while other tentatively identified genera are pathogens that have not previously been associated with wasps (e.g. *Geomyces*, which includes species that are pathogens of bats [46]). We also tentatively identified eight nematode taxa belonging to seven different genera. Some of the tentatively identified genera such as *Bursaphelenchus*, *Loa*, and *Pristionchus* were found in fig wasps [47]. Furthermore, three trypanosome genera were identified (*Eimeria*, *Toxoplasma*, and *Trypanosoma*). While none of the identified trypanosome genera have been previously associated with wasps, there are trypanosome genera that have been shown to infect hymenopterans (e.g. *Crithidia* [44]). Three tapeworm genera were also observed (*Diphyllobothrium*, *Echinococcus*, and *Taenia*), of which none has previously been associated with hymenoptera. There were no virus peptides identified.

**Fig 3:**
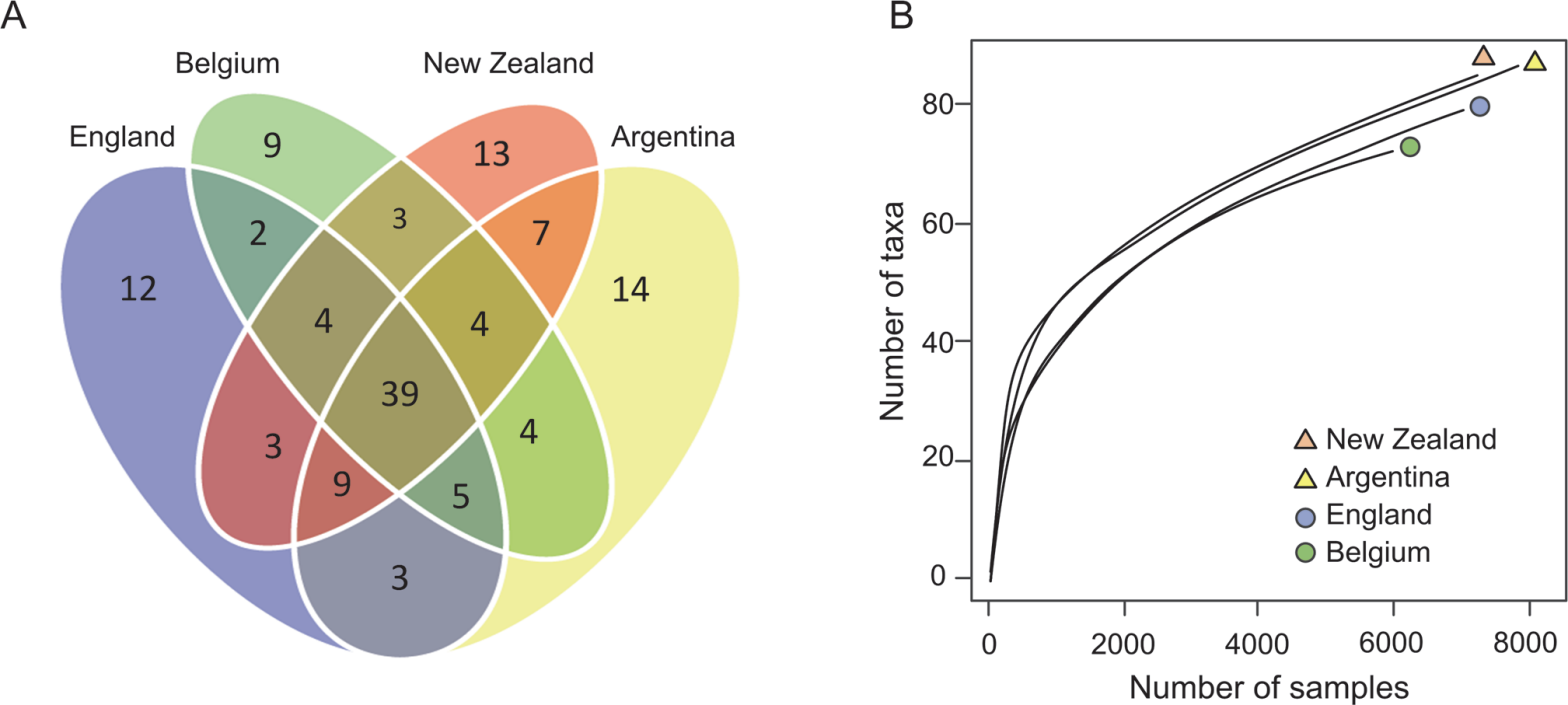
Microbial communities in wasp samples from the four countries. (A) A Venn diagram showing the overlap and distinctiveness of microbial taxa from common wasps in the native (England and Belgium) and invaded range (New Zealand and Argentina). A total of 131 peptides from distinct microbial taxa were observed. Of these 131 microbial taxa, 39 taxa were shared between all countries, but different countries had between 9–14 distinct taxa. (B) Rarefaction curves showing the similarity of microbial taxa accumulation with increasing peptides sampled.

Similar numbers of microbial taxa were observed in the native as in the invaded range (Fig. 2B). Binomial generalised linear models found no difference in microbial taxa identified using proteomics between the invaded and native ranges (*F* = 2.719, D.F. = 1, *P* = 0.099), and no difference among the four countries sampled (*F* = 1.109, D.F. = 3, *P* = 0.345). Neither model found differences between taxa (ranges: *F* = 1.366, D.F. = 3, *P* = 0.252; countries: *F* = 1.363, D.F. = 3, *P* = 0.253). Of the entire microbiome sampled from all wasp populations, 39 of the 131 taxa (or 29.8%) were common to all four countries (Fig. 3A). Many of the other microbial taxa were shared between at least two countries or more. The similarity to the nearest country was, in order, from England, Belgium, New Zealand and Argentina (i.e. the wasp microbiome in Argentina was most similar to that in New Zealand and most different from that in England), which is consistent with the phylogenetic relationships between wasps in these countries [10]. There were between nine and 14 distinct microbial taxa unique to each country (Fig. 3A). A flattening of rarefaction curves at similar levels among the four sampled populations indicated that the proteomic sampling was a fair representation and comparison of the taxonomic diversity in the populations (Fig. 3B).

### Phylogenetic comparisons using PCR methods

**Fig 4:**
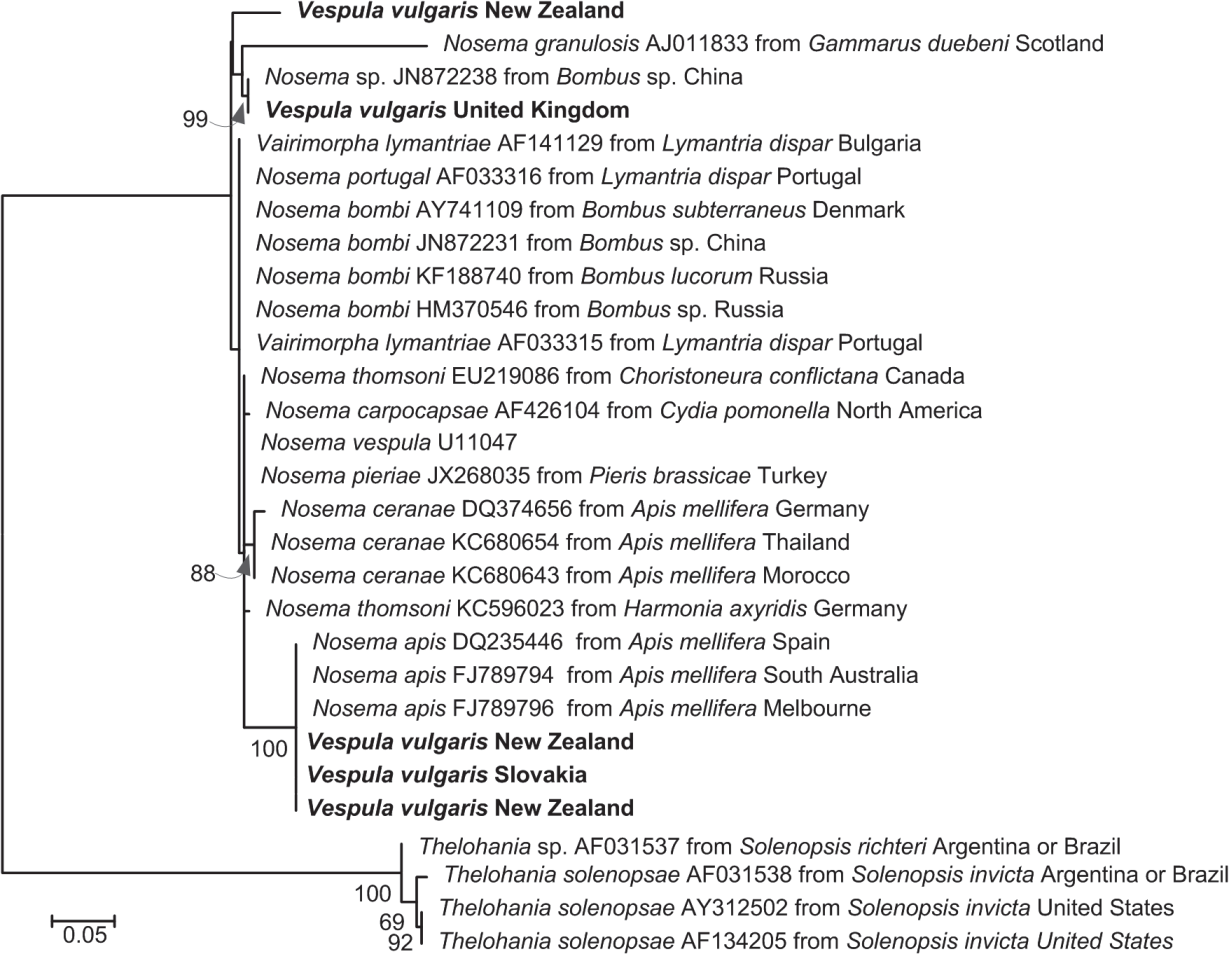
Maximum Composite Likelihood tree for putative 16S *Nosema* sequences from *Vespula vulgaris* sampled (bold) and the best matching sequences on GenBank, together with their accession numbers and sample collection locations (where available). The tree was based on 2000 bootstraps of a general time-reversible model with gamma distribution and invariant sites parameters (GTR + G(0.69) +I(0.0); lnL −928.456) in MEGA6. The estimates of levels of support shown below the nodes are bootstrap values greater than 50%. The tree is drawn to scale, with branch lengths measured in the number of substitutions per site.

Our comparison of putative *Nosema* 16S sequences from our samples of *V*. *vulgaris* to sequences on GenBank revealed identical matches to *N*. *apis* (a common pathogen of honey bees) from three samples (two from New Zealand and one from Slovakia: 100% coverage and 99% identity; Fig. 4). The sample from the United Kingdom sample matched a *Nosema* sp. sequence from *Bombus* sp. from China (100% coverage and 100% identity). The third New Zealand sample matched a number of *Nosema* species including *N*. *bombi*, *N*. *portugal* and *Vairimorpha lymantriae* sequences with 100% coverage and 94% identity (Fig. 4).

**Fig 5:**
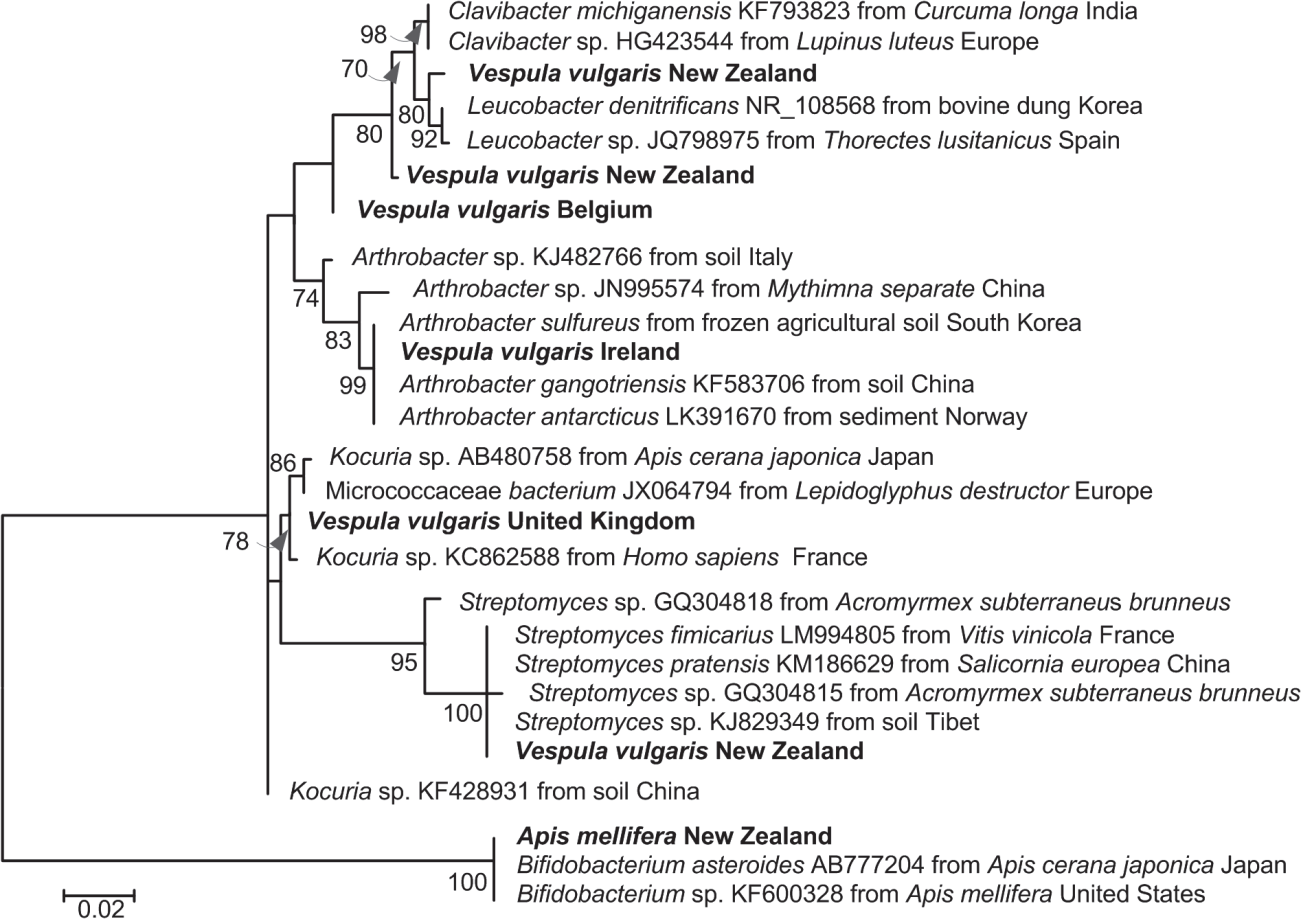
Maximum Composite Likelihood tree for putative Actinobacteria sequences from *Vespula vulgaris* sampled (bold) and the best matching sequences on GenBank, together with their accession numbers and host species. The tree was based on 2000 bootstraps of a general time-reversible model with gamma distribution and invariant sites parameters; lnL −559.13) in MEGA6. The estimates of levels of support shown below the nodes are bootstrap values greater than 50%. The tree is drawn to scale, with branch lengths measured in the number of substitutions per site. Often GenBank sequences were equally well matched to the sequences from *V*. *vulgaris* and those displayed on the tree are not exhaustive (e.g. the Ireland sample matched equally well to multiple *Arthrobacter* sp.).

16S Actinobacteria sequences from the New Zealand sample of *Apis mellifera* most closely matched (100% coverage and 100% identity) *Bifidobacterium* sp. from *A*. *cerana* and *A*. *mellifera* (Fig. 5). The Actinobacteria sequences recovered from *V*. *vulgaris* matched a variety of taxa. One New Zealand sample matched most closely to *Streptomyces* sp. (100% coverage, 100% identity), the second to *Leucobacter* sp. (99% coverage, 99% identity) and *Clavibacter* sp. (100% coverage, 98% identity), and the third to *Leucobacter denitrificans* (100% coverage, 99% identity. The Belgian sample matched most closely to *Kocuria* sp. and *Clavibacter* sp. (both 100% coverage and 98% identity). The sequence from the United Kingdom *V*. *vulgaris* sample matched to *Kocuria* sp. most closely (100% coverage, 99% identity), and the sequence from the Ireland sample matched most closely to *Arthrobacter* sp. (100% coverage, 100% identity; Fig. 5).

**Fig 6:**
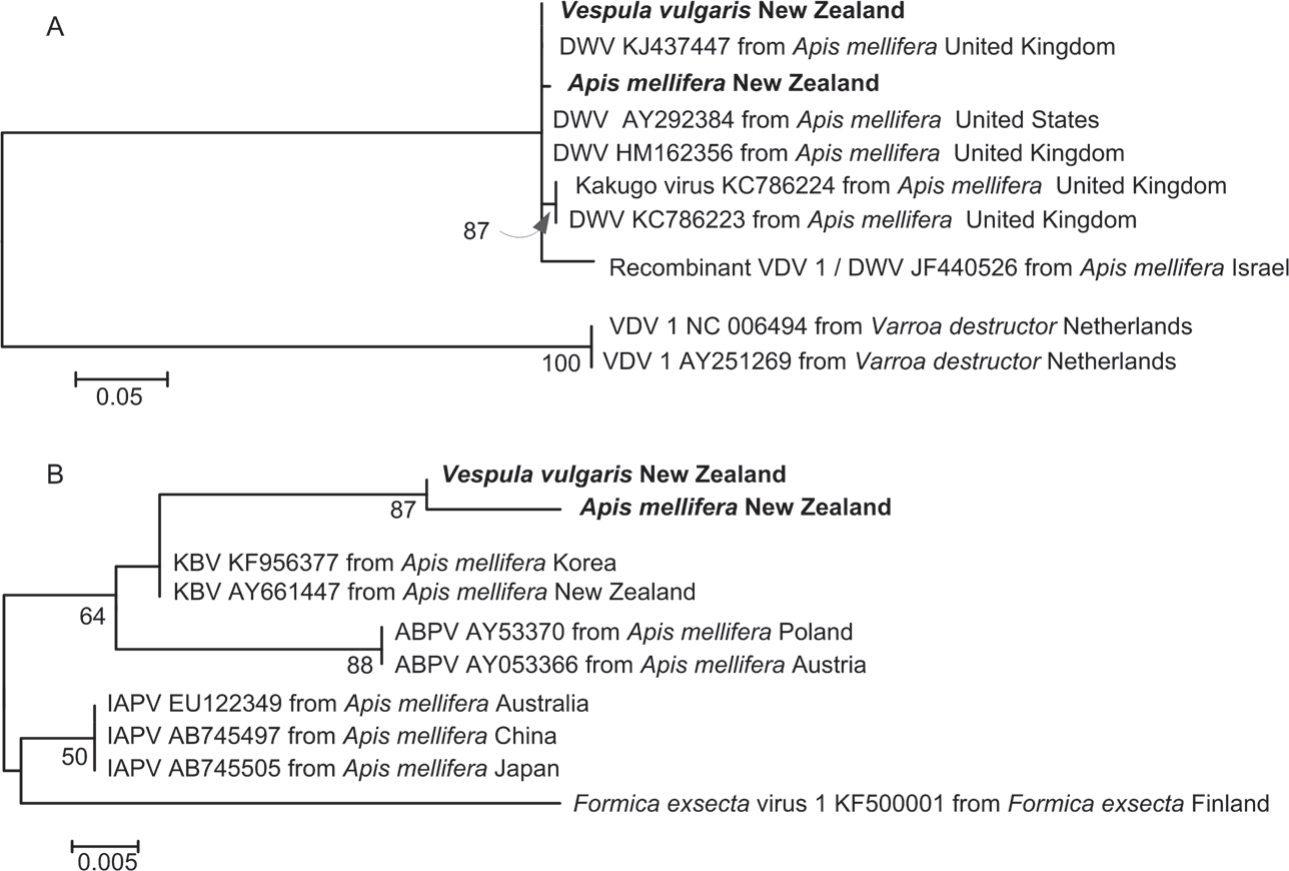
Maximum Composite Likelihood trees. (A) Putative Deformed wing virus (DWV) sequences. (B) Kashmir bee virus (KBV) sequences from *Vespula vulgaris and Apis mellifera* sampled (bold) and the best matching sequences on GenBank, together with their accession numbers and host species. The trees were based on 2000 bootstraps of a general time-reversible model with gamma distribution and invariant sites parameters (GTR + G(0.48) +I(200); lnL −550.04) for DWV and a general time-reversible model (GTR; lnL −195.50) for KBV in MEGA6. The estimates of levels of support shown below the nodes are bootstrap values greater than 50%. The trees are drawn to scale, with branch lengths measured in the number of substitutions per site.

The putatively identified Deformed wing virus sequences from New Zealand *V*. *vulgaris* samples matched Deformed wing virus sequences of *A*. *mellifera* from the United Kingdom and the United States (100% coverage, 100% identity; Fig. 6A). Sequences of Kashmir bee virus from our wasp samples most closely matched Kashmir bee virus sequences of *A*. *mellifera* from a number of locations including Korea and Australia (100% coverage, 98% identity; Fig. 6B).

## Discussion

We observed no evidence to support the enemy release hypothesis, at least in terms of the total number of microorganism taxa observed in the introduced range compared to the native range. Wasps in the introduced range had a similar prevalence of pathogen and microbial species compared to the samples from the native range, both in the historical and proteomics datasets. The pathogens observed through the proteomics methods were often identified as also pathogens of honey bee or other hymenoptera. While the total number of microbial taxa was similar between native and invaded ranges, a degree of distinctiveness was observed. Between nine and 14 taxa were unique to each country. While this could represent a sampling effect, it could also indicate that some pathogens or microbial taxa key to the density-dependent population regulation of wasps are missing from the invaded range. Different pathogens vary considerably in their virulence [48] and perhaps taxa with high virulence are absent from the invaded range. The absence of key enemies in the invaded species seems possible given the considerable fluctuation observed in population densities of wasps in countries like England [11,12], with no evidence of similar variation in abundance in the invaded range.

A recent review found significantly more evidence of a higher abundance of enemy taxa in the native versus invaded range, which supports a key component of the enemy release hypothesis [3]. Specifically within social insect communities, several studies suggest the enemy release hypothesis can play a major role in invasions and population dynamics. For example, Yang et al. [49] found red imported fire ants to have higher pathogen infections in their native range compared to the invaded range. Other studies, however, have found that invasive species can have similar or higher pathogen loads in populations of exotic species in their invaded range (e.g. [10,50]). When pathogens and microbial taxa are generalists, considerable spill-over and spill-back may occur between native and exotic taxa. These generalist pathogens and parasites might even facilitate invasion by acting as a biological weapon, especially if the pathogens are less virulent in the invasive species. Such spill-over or spill-back effects of pathogens have been referred to as the ‘enemy of my enemy’ hypothesis [51] or the ‘disease-mediated invasion’ hypothesis [52] wherein the invasive species benefits from an enemy alliance rather than from enemy release. The invasion of an exotic lady beetle and its obligate pathogen, resulting in the displacement of native species, has recently been suggested as one example of a disease mediated invasion [53]. Just how the generalist pathogens of the common wasps might interact with the community and the relative virulence of these pathogens remains to be determined.

Wasps and other hymenopteran species are susceptible and may even die from "honey bee" pathogens. Fantham & Porter [54] introduced the honey bee pathogen *Nosema apis* into wasp (*Vespula germanica*) nests which subsequently died. More recently, *N*. *ceranae* was shown to infect bumble bees shortening their lifespan and altering their behaviour [48]. Similarly, Deformed wing virus—a common virus in honey bees—can infect and significantly reduce longevity in bumble bees [55]. In our study, we confirmed the infection of wasps with *Nosema* by PCR, and a more widespread survey of Belgium, England, Argentina and New Zealand has recently shown infection rates of individual wasps of up to 54% [10]. Specific *Nosema* species matches on GenBank were to *N*. *apis* and *N*. *bombi*. The infection of wasps by these *Nosema* species from bees is entirely reasonable given the experimental work involving successful cross-infection of *Nosema* between different hymenopteran hosts [48,54,55]. These results are indicative that in the unintentional international movement of species such as wasps, or during the intentional international movement of other species such as bumble bees for pollination, pathogens of a wide range of host species (including honey bee pathogens) are also being moved.

Proteomics methods have previously been used for pathogen discovery (e.g. [22]). Authors have highlighted the importance of an appropriate protein database to limit misidentification or false discovery [25], and methodological issues including the tendency for liquid chromatography-tandem MS (LC-MS/MS) (shotgun proteomics) to identify the most abundant proteins more frequently [56]. We have attempted to avoid the analytical issues by using the most comprehensive protein sequence databases available (NCBInr and LudwigNR), comprising of all taxonomies including honey bees, wasps and pathogen proteins. We used SDS-PAGE and gel slicing to fractionate the proteins into 20 fractions prior to LC-MS/MS analysis to increase the identification of low abundance proteins. We do recognise, however, that the use of proteomics for pathogen discovery is in its early stages. The compilation of personalised sequence databases and hence the ability to recognize pathogens is considered work in progress.

The proteomics analysis failed to find conclusive evidence of any virus peptides. We consider that this is likely to be a false-negative result and a methodological limitation rather than the absence of viruses in wasp samples. Despite our approach to fractionate the samples (20 gel slices), it is clear that a more targeted mass spectrometry approach is warranted since any peptides of viral origin will be of low abundance compared with wasp house-keeping proteins such as actin and myosin. Evidence of two viruses (Deformed wing virus and Kashmir bee virus) was found in the genetic analysis. As many as a third of common wasps in the United Kingdom have been observed to be infected with Deformed wing virus [20], which is a major pathogen of bees and has been considered as the main suspect behind unexplained honey bee colonies collapsing worldwide [57]. Kashmir bee virus has previously been observed in common wasps in New Zealand [18]. Elsewhere *Vespula* sp. wasps have been known to be infected with a range of viruses commonly found in honey bees, including the Israeli acute paralysis virus, Deformed wing virus, Kashmir bee virus, Black queen cell virus, and Sacbrood virus [19]. The transmission of the viruses between species has been observed to occur via foraging in the same environment on flowers or pollen [19]. Replication of these “honey bee” viruses has been observed within a range of alternative host species including hornets [58]. Thus many of these viruses and other pathogens that were first described from honey bees, appear shared and abundant across a wide range of insect species [59]. Similarly, bees may introduce bacterial pathogens such as *Arsenophonus* spp. into the nectar of flowers, which then may be inoculated into other pollinators and nectar thieves [16,19]. This sharing of pathogens can result in a correlated prevalence of viruses and fungal species between hymenopteran species such as bumble bees and honey bees [55].

Our results are indicative that beneficial microbial taxa are also shared between wasp and bee species. The proteomics analysis indicated the presence of Actinobacteria with six taxa most closely matching PCR sequences from our samples. These bacteria are known to provide a level of resistance to pathogens of honey bees such as American foulbrood [60] and other species including paper wasps [61]. Like the viruses, many species of Actinobacteria found in honey bees are also present in floral nectar, allowing horizontal transmission between bees [62] and other nectar foraging species including wasps. The acquisition of beneficial microbial taxa from other insects may effectively offset any bottleneck loss of mutualistic microbes that could have occurred during the wasps’ invasion into their new range. Thus, it is not unexpected that wasps and other pollinators are exposed to a wide range of microbial taxa, but it is more surprising that such viruses and bacteria are able to tolerate the range of gastrointestinal and physiological environments associated with different herbivorous, omnivorous and carnivorous insect species.

Invasive social insects such as the common wasp are a major problem in many countries [63]. Our findings that suggest a lack of enemy release and generality of a pathogen fauna have major implications for their management. The likely spill-over and spill-back of pathogens and parasites that vary in their virulence between host species makes these community dynamics complicated, particularly over large scales that may have highly variable ecological communities. However, the large spatial distribution and high abundance of wasps in countries such as New Zealand makes widespread chemical control impractical and biological control more attractive [27,64]. If pathogens of wasps are not host specific and instead are shared with key ecosystem service providers like honey bees, any introduction of pathogens or attempts to encourage pathogen abundance may have unintended negative consequences. Nevertheless populations of wasps in countries like England demonstrate considerable fluctuation [10,11], which is perhaps indicative of pathogen and parasite effects, but honey bee and bumble bee populations appear to be in sufficient abundance to provide ecosystem services. Future work towards understanding reasons for the population fluctuations of wasps in the native range should focus on species- or genera-specific pathogens or microbial interactions.

## Supporting Information

S1 Table A list of microorganisms previously identified from *Vespula vulgaris* and *V*. *germanica*, in their native and introduced range. Note that all previous *V*. *vulgaris* observations in North America were assumed to be the native species *V*. *alascensis* [28]. Within Rose et al. [18] several observations were identified only to the genus level, such as “*Streptococcus* sp.”. We treated all sp. identification as a new species, which may have overestimated the microbial community (in comparison, not using these records may underestimate the microbial community). We also note that these are “possible” pathogens and some of the taxa identified by may not be harmful or may even be mutualistic. Any determination of pathogenicity would require experimental work.
(DOCX)

S2 Table Proteins and organisms identified in wasps in the native (England and Belgium) and invaded (New Zealand and Argentina) range. The full dataset of mass spectrometry proteomics data have been deposited to the ProteomeXchange Consortium (http://proteomecentral.proteomexchange.org) [33] via the PRIDE partner repository with the dataset identifiers PXD001586 and DOI W50heXfw.
(XLSX)

## References

[1] Keane RM, Crawley MJ. Exotic plant invasions and the enemy release hypothesis. Trends Ecol Evol. 2002;17: 164–170.

[2] Torchin ME, Lafferty KD, Dobson AP, McKenzie VJ, Kuris AM. Introduced species and their missing parasites. Nature 2003;421: 628–630. 12571595 10.1038/nature01346

[3] Heger T, Jeschke JM. The enemy release hypothesis as a hierarchy of hypotheses. Oikos 2014;123: 741–750.

[4] Archer ME. A Key to the world species of the Vespinae (Hymenoptera). Res Monogr Coll Ripon St. John 1989;2: 1–41.

[5] Dvorák L. Social wasps (Hymenoptera: Vespidae) trapped with beer in European forest ecosystems. Acta Mus Morav. 2007;92: 181–204.

[6] Moller H, Tilley JAV, Thomas BW, Gaze PD. Effect of introduced social wasps on the standing crop of honeydew in New Zealand beech forests. N Z J Zool. 1991;18: 171–180.

[7] Beggs JR, Toft RJ, Malham JP, Rees JS, Tilley JAV, Moller H, et al The difficulty of reducing introduced wasp (*Vespula vulgaris*) populations for conservation gains. N Z J Ecol. 1998;22: 55–63. 9599853

[8] Toft RJ, Rees JS. Reducing predation of orb-web spiders by controlling common wasps (*Vespula vulgaris*) in a New Zealand beech forest. Ecol Entomol. 1998;23: 90–95.

[9] Gardner-Gee R, Beggs JR. Invasive wasps, not birds, dominate in a temperate honeydew system. Austral Ecol. 2012;38: 346–354.

[10] Lester PJ, Gruber MAM, Brenton-Rule EC, Archer E, Corley JC, Dvořák L, et al Determining the origin of invasions and demonstrating a lack of enemy release from microsporidian pathogens in common wasps (Vespula vulgaris). Divers Distrib. 2014;8: 964–974.

[11] Archer ME. Successful and unsuccessful development of colonies of Vespula vulgaris (Linn.) (Hymenoptera: Vespidae). Ecol Entomol. 1981;6: 1–10.

[12] Archer ME. Population dynamics of the social wasps *Vespula vulgaris* and *Vespula germanica* in England. J Anim Ecol. 1985;54: 473–485.

[13] Barlow ND, Beggs JR, Barron MC. Dynamics of common wasps in New Zealand beech forests: a model with density dependence and weather. J Anim Ecol. 2002;71: 663–671.

[14] Evans JD, Schwarz RS. Bees brought to their knees: microbes affecting honey bee health. Trends Microbiol. 2011;19: 614–620. 10.1016/j.tim.2011.09.003 22032828

[15] Anderson KE, Sheehan TH, Eckholm BJ, Mott BM, De Grandi-Hoffman G. An emerging paradigm of colony health: microbial balance of the honey bee and hive (Apis mellifera). Insectes Soc. 2011;58: 431–444.

[16] Aizenberg-Gershtein Y, Izhaki I, Halpern M. Do honeybees shape the bacterial community composition in floral nectar? PLoS One 2013;8: e67556 10.1371/journal.pone.0067556 23844027 PMC3701072

[17] Fries I, Camazine S. Implications of horizontal and vertical pathogen transmission for honey bee epidemiology. Apidologie 2001;32: 199–214.

[18] Rose EAF, Harris RJ, Glare TR. Possible pathogens of social wasps (Hymenoptera: Vespidae) and their potential as biological control agents. N Z J Zool. 1999;26: 179–190.

[19] Singh R, Levitt AL, Rajotte EG, Holmes EC, Ostiguy N, vanEngelsdorp W, et al RNA viruses in Hymenopteran pollinators: evidence of inter-taxa virus transmission via pollen and potential impact on non-apis hymenopteran species. PLOS One 2010;5: e14357 10.1371/journal.pone.0014357 21203504 PMC3008715

[20] Evison SEF, Roberts KE, Laurenson L, Pietravalle S, Hui J, Biesmeijer JC, et al Pervasiveness of parasites in pollinators. PLoS One 2012;7: e30641 10.1371/journal.pone.0030641 22347356 PMC3273957

[21] Aebersold R, Mann M. Mass spectrometry-based proteomics. Nature 2003;422: 198–207. 12634793 10.1038/nature01511

[22] Bromenshenk JJ, Henderson CB, Wick CH, Stanford MF, Zulich AW, Jabbour RE, et al Iridovirus and microsporidian linked to honey bee colony decline. PLoS One 2010;5: e13181 10.1371/journal.pone.0013181 20949138 PMC2950847

[23] Keller A, Nesvizhskii AI, Kolker E, Aebersold R. Empirical statistical model to estimate the accuracy of peptide identifications made by MS/MS and database search. Anal Chem 2002;74: 5383–5392. 12403597 10.1021/ac025747h

[24] Jabbour RE, Deshpande SV, Wade MM, Stanford MF, Wick CH, Zulich AW, et al Double-blind characterization of non-genome-sequenced bacteria by mass spectrometry-based proteomics. Appl Environ Microbiol. 2010;76: 3637–3644. 10.1128/AEM.00055-10 20363779 PMC2876470

[25] Knudsen GM, Chalkley RJ. The effect of using an inappropriate protein database for proteomic data analysis. PLoS One 2011;6: e20873 10.1371/journal.pone.0020873 21695130 PMC3114852

[26] Boerjan B, Cardoen D, Verdonck R, Caers J, Schoofs L. Insect omics research coming of age. Can J Zool. 2012;90: 440–455.

[27] Lester PJ, Brown SDJ, Edwards ED, Holwell GI, Pawson SM, Ward DF, et al Critical issues facing New Zealand entomology. N Z Entomol. 2014;37: 1–13.

[28] Carpenter TM, Glare TJ. Misidentification of Vespula alascensis as V. vulgaris in North America (Hymenoptera: Vespidae; Vespinae). Am Mus Novit. 2010;3690: 1–7.

[29] Bates D, Maechler M, Bolker B, Walker S. lme4: Linear mixed-effects models using Eigen and S4. R package version 1.1–7. 2014. Available: http://CRAN.R-project.org/package=lme4.

[30] R Development Core Team. R: a language and environment for statistical computing 2014; R Foundation for Statistical Computing, Vienna, Austria Available: http://www.R-project.org.

[31] Peng LF, Kapp EA, McLauchlan D, Jordan TW. Characterization of the Asia Oceania human proteome organisation membrane proteomics initiative standard using SDS-PAGE shotgun proteomics. Proteomics 2011;11: 4376–4384. 10.1002/pmic.201100169 21887821

[32] Nesvizhskii AI, Keller A, Kolker E, Aebersold R. A statistical model for identifying proteins by tandem mass spectrometry. Anal Chem. 2003;75: 4646–4658. 14632076 10.1021/ac0341261

[33] Vizcaino JA, Deutsch EW, Wang R, Csordas A, Reisinger F, Rios D, et al ProteomeXchange provides globally coordinated proteomics data submission and dissemination. Nature Biotechnology 2014;32: 223–226. 10.1038/nbt.2839 24727771 PMC3986813

[34] Oksanen J, Blanchet FG, Kindt R, Legendre P, Minchin PR, O’Hara RB, et al. vegan: Community Ecology Package. 2013;R package version 2.0–7. Available: http://CRAN.R-project.org/package=vegan.

[35] Jacobs J. Individual based rarefaction using R-package; 2011. Available: http://www.jennajacobs.org/R/rarefaction.html.

[36] Stach JEM, Maldonado LA, Ward AC, Goodfellow M, Bull AT. New primers for the class Actinobacteria: application to marine and terrestrial environments. Environ Microbiol. 2003;5: 828–841. 14510836 10.1046/j.1462-2920.2003.00483.x

[37] Baker MD, Vossbrinck CR, Didier ES, Maddox JV, Shadduck JA. Small subunit ribosomal DNA phylogeny of various microsporidia with emphasis on AIDS related forms. J Eukaryot Microbiol. 1995;42: 564–570. 7581329 10.1111/j.1550-7408.1995.tb05906.x

[38] Lanzi G, de Miranda JR, Boniotti MB, Cameron CE, Lavazza A, Capucci L, et al Molecular and biological characterization of Deformed wing virus of honeybees (*Apis mellifera* L.). J Virol. 2006;80: 4998–5009. 16641291 10.1128/JVI.80.10.4998-5009.2006 PMC1472076

[39] Francis RM, Kryger P. Single assay detection of Acute bee paralysis virus, Kashmir bee virus and Israeli acute paralysis virus. J Apic Sci. 2012;56: 137–146.

[40] Tamura K, Stecher G, Peterson D, Filipski A, Kumar S. MEGA6: Molecular Evolutionary Genetics Analysis Version 6.0. Mol Biol Evol. 2013;30: 2725–2729. 10.1093/molbev/mst197 24132122 PMC3840312

[41] Tavaré S. Some probabilistic and statistical problems in the analysis of DNA sequences. Lect Math Life Sci. 1986;17: 57–86.

[42] Gu X, Li WH. A general additive distance with time-reversibility and rate variation among nucleotide sites. Proc Natl Acad Sci U S A. 1996;93: 4671–4676. 8643462 10.1073/pnas.93.10.4671 PMC39337

[43] Kristof LJ. Über einheimische, gesellig lebende wespen und ihren nestbau. Mitt Naturwiss Ver Steiermark. 1879;15: 38–49

[44] Urdaneta-Morales S. Some biological characteristics of a *Crithidia* species from the yellow-jacket wasp, *Vespula squamosa* (Hymenoptera: Vespidae). Rev Bras Biol. 1983;43: 409–412.

[45] Reeson AF, Jankovic T, Kasper ML, Rogers S, Austin AD. Application of 16S rDNA-DGGE to examine the microbial ecology associated with a social wasp *Vespula germanica* . Insect Mol Biol. 2003;12: 85–91. 12542639 10.1046/j.1365-2583.2003.00390.x

[46] Blehert DS, Hicks AC, Behr M, Meteyer CU, Berlowski-Zier BM, Buckles EL, et al Bat white-nose syndrome: an emerging fungal pathogen? Science 2009;323: 227–227. 10.1126/science.1163874 18974316

[47] Kanzaki N, Tanaka R, Giblin-Davis RM, Davies KA. New plant-parasitic nematode from the mostly mycophagous Genus *Bursaphelenchus* discovered inside figs in Japan. PLOS ONE 2014;9: e99241 10.1371/journal.pone.0099241 24940595 PMC4062417

[48] Graystock P, Yates K, Darvill B, Goulson D, Hughes WOH. Emerging dangers: deadly effects of an emergent parasite in a new pollinator host. J Invertebr Pathol. 2013;114: 114–119. 10.1016/j.jip.2013.06.005 23816821

[49] Yang CC, Yu YC, Valles SM, Oi DH, Chen YC, Shoemaker D, et al Loss of microbial (pathogen) infections associated with recent invasions of the red imported fire ant *Solenopsis invicta* . Biol Invasions 2010;12: 3307–3318.

[50] Jones CM, Brown MJF. Parasites and genetic diversity in an invasive bumblebee. J Anim Ecol. 2014;83: 1428–1440. 24749545 10.1111/1365-2656.12235 PMC4235342

[51] Colautti RI, Ricciardi A, Grigorovich IA, MacIsaac HJ. Is invasion success explained by the enemy release hypothesis? Ecol Lett. 2004;7: 721–733.

[52] Strauss A, White A, Boots M. Invading with biological weapons: the importance of disease-mediated invasions. Funct Ecol. 2012;26: 1249–1261.

[53] Vilcinskas A, Stoecker K, Schmidtberg H, Rohrich CR, Vogel H. Invasive harlequin ladybird carries biological weapons against native competitors. Science 2013;340: 862–863. 10.1126/science.1234032 23687046

[54] Fantham HB, Porter A. The pathogenicity of *Nosema apis* to insects other than hive bees. Ann Trop Med Parasitol. 1913;8: 623–38.

[55] Furst MA, McMahon DP, Osborne JL, Paxton RJ, Brown MJF. Disease associations between honeybees and bumblebees as a threat to wild pollinators. Nature 2014;506: 364–366. 10.1038/nature12977 24553241 PMC3985068

[56] Foster LJ. Interpretation of data underlying the link between Colony Collapse Disorder (CCD) and an invertebrate iridescent virus. Mol Cell Proteomics 2011;10: M110.006387. 10.1074/mcp.M110.006387 PMC3047166 21364086

[57] Schroeder DC, Martin SJ. Deformed wing virus. The main suspect in unexplained honeybee deaths worldwide. Virulence 2012;3: 589–591. 10.4161/viru.22219 23154287 PMC3545936

[58] Yanez O, Zheng HQ, Hu FL, Neumann P, Dietemann V. A scientific note on Israeli acute paralysis virus infection of Eastern honeybee *Apis cerana* and vespine predator *Vespa velutina* . Apidologie 2012;43: 587–589.

[59] Levitt AL, Singh R, Cox-Foster DL, Rajotte E, Hoover K, Ostiguy N, et al Cross-species transmission of honey bee viruses in associated arthropods. Virus Res 2013;176: 232–240. 10.1016/j.virusres.2013.06.013 23845302

[60] Forsgren E, Olofsson TC, Vasquez A, Fries I. Novel lactic acid bacteria inhibiting *Paenibacillus larvae* in honey bee larvae. Apidologie 2010;41: 99–108.

[61] Madden AA, Grassetti A, Soriano JAN, Starks PT. Actinomycetes with antimicrobial activity isolated from paper wasp (Hymenoptera: Vespidae: Polistinae) nests. Environ Entomol. 2013;42: 703–710. 10.1603/EN12159 23905732

[62] Anderson KE, Sheehan TH, Mott BM, Maes P, Snyder L, Schwan MR, et al Microbial ecology of the hive and pollination landscape: bacterial associates from floral nectar, the alimentary tract and stored food of honey bees (Apis mellifera). PLoS One 2013;8: e83125 10.1371/journal.pone.0083125 24358254 PMC3866269

[63] Beggs JR, Brockerhoff EG, Corley JC, Kenis M, Masciocchi M, Muller F, et al Ecological effects and management of invasive alien Vespidae. Biocontrol 2011;56: 505–526.

[64] Lester PJ, Beggs JR, Brown RL, Edwards ED, Groenteman R, Toft RJ, et al The outlook for control of New Zealand’s most abundant, widespread and damaging invertebrate pests: social wasps. N Z Sci Rev. 2013;70: 56–62.

