# Supplementary material for: No Evidence of Enemy Release in Pathogen and Microbial Communities of Common Wasps (Vespula vulgaris) in Their Native and Introduced Range
Source: PLoS One. 2015 Mar 23;10(3):e0121358. doi: 10.1371/journal.pone.0121358 (PMC4370511; doi:10.1371/journal.pone.0121358)
Supplement: S1 Table — Note that all previous V. vulgaris observations in North America were assumed to be the native species V. alascensis [28]. Within Rose et al. [18] several observations were identified only to the genus level, such as “Streptococcus sp.”. We treated all sp. identification as a new species, which may have overestimated the microbial community (in comparison, not using these records may underestimate the microbial community). We also note that these are “possible” pathogens and some of the taxa identified by may not be harmful or may even be mutualistic. Any determination of pathogenicity would require experimental work. (DOCX) [file pone.0121358.s001.docx]

Electronic supplementary material:

# **No evidence of enemy release in pathogen and microbial communities of common wasps (*Vespula vulgaris*) in their native and introduced range**

P.J. Lester^1*^, P.J. Bosch^2,3^, M.A.M. Gruber^1^, E.A. Kapp^4^, L. Peng^2^, E.C. Brenton-Rule^1^, J. Buchanan^1^, W.L. Stanislawek^5^, M. Archer^6^, J.C. Corley^7^, M. Masciocchi^7^, A. Van Oystaeyen^8^, and T. Wenseleers^8^

**Table S1:** A list of microorganisms previously identified from *Vespula vulgaris* and *V. germanica*, in their native and introduced range. Note that all previous *V. vulgaris* observations in North America were assumed to be the native species *V. alascensis* (Carpenter & Glare 2010). Within Rose et al. (1999) several observations were identified only to the genus level, such as “*Streptococcus* sp.”. We treated all sp. identification as a new species, which may have overestimated the microbial community (in comparison, not using these records may underestimate the pathogen community). We also note that these are “possible” pathogens and some of the taxa identified by may not be harmful or may even be mutualistic. Any determination of pathogenicity would require experimental work.

| **Group & Taxa** | **Species** | **Country** | **Range** | **Reference** |
| --- | --- | --- | --- | --- |
|  |  |  |  |  |
| **Bacteria** |  |  |  |  |
| *Bacillus cereus* sp. | *V. germanica* | Australia | Invaded | Reeson et al. (2003) |
| *Enterococcus* sp. | *V. germanica* | Australia | Invaded | Reeson et al. (2003) |
| *Lactobacillus kunkeei* | *V. germanica* | Australia | Invaded | Reeson et al. (2003) |
| *Lactococcus lactis* | *V. germanica* | Australia | Invaded | Reeson et al. (2003) |
| *Leuconostoc* sp. | *V. germanica* | Australia | Invaded | Reeson et al. (2003) |
| *Rickettsiella grylli* | *V. germanica* | Australia | Invaded | Reeson et al. (2003) |
| *Serratia marcescens* | *V. germanica* | New Zealand | Invaded | Glare et al. (1993) |
| *Vagococcus entomophilus* | *V. vulgaris* | Czech Republic | Native | Killer et al. (2014) |
| *Wolbachia* sp. | *V. germanica & V. vulgaris* | UK | Native | Evison et al. (2012) |
|  |  |  |  |  |
| **Fungi** |  |  |  |  |
| *Alternaria* sp. | *V. vulgaris* | England | Native | Glare et al. unpubl. (cited in Rose et al. 1999) |
| *Ascosphaera* sp. | *V. germanica & V. vulgaris* | UK | Native | Evison et al. (2012) |
| *Aspergillus flavus* | *V. vulgaris* | New Zealand | Invaded | Harris et al. (2000) |
| *Aspergillus flavus* | *V. vulgaris* | New Zealand | Invaded | Glare et al. (1996) |
| *Beauveria bassiana* | *V. vulgaris* | New Zealand | Invaded | Harris et al. (1996) |
| *Beauveria bassiana* | *Vespula sp.* | New Zealand | Invaded | Barker et al. (1991) |
| *Beauveria bassiana* | *V. germanica* | New Zealand | Invaded | Wigley & Dhana (1988) |
| *Beauveria brongniartii* | *V. germanica* | New Zealand | Invaded | Laird (1991) |
| *Botrytis cinerea* | *V. germanica* | France | Native | Acolat (1953) |
| *Candida davenportii* | *V. vulgaris* | UK | Native | Stratford et al. (2002) |
| *Cordyceps ditmari* | *V. vulgaris* | Europe | Native | Petch (1932) |
| *Hirsutella saussurei* | *Vespula sp.* | New Zealand | Invaded | Glare et al. unpubl. (cited in Rose et al. 1999) |
| *Hirsutella* sp. | *V. germanica* | New Zealand | Invaded | Wigley & Dhana (1988) |
| *Nosema* spp. (2 species) | *V. germanica & V. vulgaris* | New Zealand | Invaded | Wigley & Scotti unpubl. (cited in Rose et al. 1999) |
| *Nosema* sp. | *V. germanica & V. vulgaris* | UK | Native | Evison et al. (2012) |
| *Nosema* sp. | *V. vulgaris* | Native range | Native | Lester et al. (2014) |
| *Nosema* sp. | *V. germanica* | Australia | Invaded | Anderson (cited in Rose et al. 1999) |
| *Paecilomyces farinosus* | *V. germanica* | New Zealand | Invaded | Landcare Research (1982) (cited in Rose et al. 1999) |
| Unidentified microsporidian | *V. germanica & V. vulgaris* | New Zealand | Invaded | Wigley & Dhana (1988) |
|  |  |  |  |  |
| **Nematodes** |  |  |  |  |
| *Pheromermis pachysoma* | *V. vulgaris* | Austria | Native | Kristof (1879) |
| *Pheromermis pachysoma* | *V. vulgaris* | England | Native | Baird (1853) in Poinar et al. (1976) |
| *Pheromermis pachysoma* | *V. germanica* | England | Native | von Linstow (1905) |
| *Pheromermis pachysoma* | *V. vulgaris* | England | Native | Beck (1937) |
| *Pheromermis pachysoma* | *V. germanica* | Germany | Native | Kloft (1951) |
| *Pheromermis pachysoma* | *V. germanica* | Germany | Native | Gauss (1970) |
| *Pheromermis pachysoma* | *V. vulgaris* | England | Native | Waterson & Baylis (1930) in Poinar et al. (1976) |
| *Pheromermis pachysoma* | *V. germanica & V. vulgaris* | England | Native | Blackith & Stevenson (1958) |
| *Pheromermis pachysoma* | *V. germanica & V. vulgaris* | Austria | Native | Kaiser (1987) |
| *Sphaerularia bombi* | *V. vulgaris* | England | Native | Cobbold (1888) |
| *Sphaerularia bombi* | *V. vulgaris* | Germany | Native | Stammer (1934) |
| *Steinernema carpocapsae* | *V. germanica* | Australia | Invaded | Bedding (1984) |
|  |  |  |  |  |
| **Viruses** |  |  |  |  |
| Black queen cell virus | *V. vulgaris* | UK | Native | Evison et al. (2012) |
| Cricket paralysis virus | *V. germanica* | New Zealand | Invaded | Wigley & Dhana (1988) |
| Deformed wing virus | *V. germanica & V. vulgaris* | UK | Native | Evison et al. (2012) |
| Deformed wing virus | *V. germanica & V. vulgaris* | UK | Native | Evison et al. (2012) |
| Kashmir bee virus | *V. germanica* | New Zealand | Invaded | Wigley & Dhana (1988) |
| Kashmir bee virus | *V. germanica* | Australia | Invaded | Ward et al. (2007) |
| Sacbrood virus | *V. vulgaris* | UK | Native | Evison et al. (2012) |
|  |  |  |  |  |

**References**

Acolat L (1953) Les materiaux des nids de guepes. Ann Sci Univ Besancon 8: 39-43.

Akhurst RJ (1980) Morphological and functional dimorphism in *Xenorhabdus* spp., bacteria symbiotically associated with insect pathogenic nematodes *Neoaplectana* and *Heterorhabditis*. J Gen Microbiol 121: 303-309.

Barker GM, Goh HH, Lyons SN, Addison, PJ (1991) Comparative pathogenicity to Argentine stem weevil of *Beauveria bassiana* from various hosts. Proceedings of the 44th New Zealand Weed and Pest Control Conference. Pp. 214-215.

Beck R (1937) *Mermis* thread worm (Nematode) in wasp (*Vespa vulgaris*). Entomologist's Record and Journal of Variation 49: 65.

Bedding RA (1984) Nematode parasites of Hymenoptera. In: Nickle, W. R. ed. Plant and insect nematodes. New York, Marcel Dekker. Pp. 755-795.

Blackith RE, Stevenson JH (1958) Autumnal populations of wasp's nests. Insect Soc 5: 347-352.

Cobbold TS (1888) On *Simondsia paradoxa* and on its probable affinity with *Sphaerularia bombi*. Trans. Linnean Soc. London 2: 357-361

Evison SEF, Roberts KE, Laurenson L, Pietravalle S, Hui J, Biesmeijer JC, Smith JE, Budge G, Hughes WOH (2012) Pervasiveness of parasites in pollinators. PLoS One 7: e30641.

Gauss R (1970) Beitrag ziir Kenntnis von Parasitoiden bei aculeaten Hymenopteren. Z Angew Zool 65: 239-244.

Glare TR, Harris RJ, Donovan BJ (1996) *Aspergillus flavus* as a pathogen of wasps, *Vespula* spp., in New Zealand. NZ J Zool 23: 339-344.

Glare TR, O'Callaghan M, Wigley PJ (1993) Checklist of naturally occurring entomopathogenic microbes and nematodes in New Zealand. NZ J Zool 20: 95-120.

Harris RJ, Harcourt SJ, Glare TR, Rose EAF, Nelson TJ (2000) Susceptibility of *Vespula vulgaris* (Hymenoptera: Vespidae) to generalist entomopathogenic fungi and their potential for wasp control. J Invertebr Pathol 75: 251-258.

Killer J, Svec P, Sedlacek I, Cernohlavkova J, Benada O, Hroncova Z, Havlik J, Vlkova E, Rada V, Kopecny J, Kofronova O (2014) *Vagococcus entomophilus* sp nov., from the digestive tract of a wasp (*Vespula vulgaris*). Int J Syst Evol Micr 64: 731-737.

Kloft W (1951) Pathologische Untersuchungen an einem Wespen-Weibchen, infiziert durch einen Gordioiden (Nematomorpha). Z Parasitenk 15: 134-147.

Kristof LJ (1879) Ueber einheimische, gesellig lebende wespen und ihren nestbau. Mitt Naturwiss Ver Steiermark 15: 38-49.

Laird M (1991) Australasian Invertebrate Pathology Working Group Newsletter XI: 3-4.

Lester PJ, Gruber MAM, Brenton-Rule EC, Archer M, Corley JC, Dvorak L, Masciocchi M, Van Oystaeyen A (2014) Determining the origin of invasions and demonstrating a lack of enemy release from microsporidian pathogens in common wasps (*Vespula vulgaris*). Divers Distrib 20: 964-974.

Petch T (1932) Cordyceps on Hymenoptera. T Brit Mycol Soc 16: 219-221.

Poinar GO, Lane RS, Thomas GM (1976) Biology and redescription of *Pheromermis pachysoma* (von Linstow) n. gen., n. comb. (Nematoda: Mermithidae), a parasite of yellowjackets (Hymenoptera: Vespidae). Nematologica 22: 360-370.

Reeson AF, Jankovic T, Kasper ML, Rogers S, Austin AD (2003) Application of 16S rDNA-DGGE to examine the microbial ecology associated with a social wasp *Vespula germanica*. Insect Mol Biol 12: 85-91.

Rose EAF, Harris RJ, Glare TR (1999) Possible pathogens of social wasps (Hymenoptera: Vespidae) and their potential as biological control agents. NZ J Zool 26: 179-190.

Stammer HI (1934) Die nematoden als kommensalen und parasiten der insekten. Verh Dtsch Zool Ges 38: 195-206.

Stratford M, Bond CJ, James SA, Roberts IN, Steels H (2002) *Candida davenportii* sp nov., a potential soft-drinks spoilage yeast isolated from a wasp. Int J Syst Evol Micr 52: 1369-1375.

Von Linstow O (1905) Helminthologische Beobachtungen. Arch Mikrosk Anat 66: 355-366.

Ward L, Waite R, Boonham N, Fisher T, Pescod K, Thompson H, Chantawannakul P, Brown M (2007) First detection of Kashmir bee virus in the UK using real-time PCR. Apidologie 38: 181-190.

Wigley PJ, Dhana S (1988) Prospects for microbial control of the social wasps, *Vespula germanica* and *V. vulgaris*. Australasian Invertebrate Pathology Working Group newsletter IX: 17.
